# Long-Read Sequencing Identifies Mosaic Sequence Variations in Friedreich’s Ataxia-GAA Repeats

**DOI:** 10.3390/ijms26114969

**Published:** 2025-05-22

**Authors:** Joohyun Park, Claudia Dufke, Zofia Fleszar, Michael Schlotterbek, Elena Buena-Atienza, Lara G. Stühn, Caspar Gross, Marc Sturm, Stephan Ossowski, Ludger Schöls, Olaf Riess, Tobias B. Haack

**Affiliations:** 1 Institute of Medical Genetics and Applied Genomics, University of Tübingen, 72076 Tübingen, Germany; 2 Department of Neurodegenerative Diseases, Center for Neurology and Hertie-Institute for Clinical Brain Research, University of Tübingen, 72076 Tübingen, Germany; 3 NGS Competence Center Tübingen, 72076 Tübingen, Germany; 4 Center for Rare Diseases, University of Tübingen, 72076 Tübingen, Germany; 5 German Center of Neurodegenerative Diseases (DZNE), 72076 Tübingen, Germany

**Keywords:** long-read sequencing, FRDA, Friedreich’s ataxia, Oxford Nanopore Technologies, repeat expansion

## Abstract

Friedreich’s ataxia (FRDA) is an autosomal recessive neurodegenerative disorder characterized by ataxia, sensory loss and pyramidal signs. While the majority of FRDA cases are caused by biallelic GAA trinucleotide repeat expansions in intron 1 of FXN, there is a subset of patients harboring a heterozygous pathogenic small variant compound-heterozygous with a GAA repeat expansion. We report on the diagnostic journey of a 21-year-old patient who was clinically suspected of having FRDA at the age of 12 years. Genetic testing included fragment analysis, gene panel analysis and exome sequencing, which only detected one pathogenic heterozygous missense variant (c.389 G>T,p.Gly130Val) in *FXN*. Although conventional repeat analyses failed to detect GAA expansions in our patient, subsequent short-read genome sequencing (GS) indicated a potential GAA repeat expansion. This finding was confirmed by long-read GS, which in addition revealed a complex pattern of interruptions. Both large and small GAA expansions with divergent interruptions containing G, A, GA, GAG and/or GAAG sequences were present within one allele, indicating mosaic sequence variations. Our findings underscore the complexity of repeat expansions which can exhibit both interruptions and somatic instability. We also highlight the utility of long-read GS in unraveling intricate genetic profiles, ultimately contributing to more accurate diagnoses in clinical practice.

## 1. Introduction

Friedreich’s ataxia (FRDA) is an autosomal recessive, neurodegenerative disorder characterized by various clinical manifestations, including gait and limb ataxia, dysarthria, sensory loss in lower limbs and pyramidal signs [1,2]. Hypertrophic cardiomyopathy is present in approximately 60% and diabetes mellitus in approximately 10–30% of FRDA patients. Less common clinical features include kyphoscoliosis, pes cavus, optic atrophy and hearing loss [2,3]. The majority of patients (96%) with FRDA are homozygous for the expansion of GAA trinucleotide short tandem repeats (STRs) in intron 1 of the *FXN* gene in chromosome 9q21.11. Normal alleles vary between 5 and 35 GAA repeats, and more than 66 GAA repeats are considered disease-causing. In most cases, more than 600 GAA repeats have been observed, and the number can range up to 1700 repeats [2,3]. The size of the expansion strongly correlates with disease progression and severity of disease, and inversely with age of onset [1,2]. While the size of smaller GAA repeat expansions and the amount of residual frataxin is related to age of onset and disease severity, it only accounts for approximately 45% of clinical variability [4]. FRDA usually occurs before the age of 25 years (mean 10–15 years); however, disease onset can also vary between 5 and 40 years, and several cases with atypical clinical presentation and very late manifestations (>40 years) have been reported as well [5,6,7]. A small fraction of patients (4%) have an expanded GAA repeat in one *FXN* allele and a pathogenic small variant or a deletion in the other allele [3,8]. One consanguineous family with Charcot–Marie–Tooth harbored a biallelic missense variant in *FXN* [9]. To date, there have been no reported cases with disease-causing small variants in both *FXN* alleles in FRDA patients [3,8].

*FXN* encodes for a mitochondrial iron chaperone protein, frataxin, that is ubiquitously expressed [10]. GAA expansions within the *FXN* gene induce locus-specific epigenetic modifications, revealing a characteristic pattern exhibiting hypermethylation in the upstream and hypomethylation in the downstream region of GAA repeats within intron 1 [10]. This specific epigenetic signature contributes to transcriptional gene silencing, as indicated by the decreased FXN expression observed in affected patients [7,10]. GAA expansions are known to be genetically unstable and thus susceptible to somatic expansions as well as contractions [11]. The repeat length may also undergo annual fluctuations within the same tissue; for instance, in lymphocytes, an increase of up to nine repeats has been observed per year [11]. Interruptions of the expanded repeats with different sequences such as GAAGAG, GAAGGA or GAAGAAAA have been noted to challenge the molecular diagnosis of FRDA [7,12]. The impact of such complex interruptions in GAA repeats on the age of onset and disease severity remains debated, with studies showing varied outcomes [7,12].

In this paper, we report a FRDA patient harboring a heterozygous pathogenic missense variant, in whom a GAA expansion could not be detected by conventional repeat analysis utilizing triple-repeat-primed PCR (TR-PCR) and long-range PCR (LR-PCR). Through long-read genome sequencing (GS), both large GAA expansions and small expansions with complex interruptions were identified in one allele indicative of somatic mosaicism, ultimately confirming the diagnosis of FRDA.

## 2. Results

### 2.1. Case Description

The 21-year-old German female reported that gait difficulties began at the age of 9 years and have slowly progressed since then. Initially, she noted “dragging” of both feet on the ground, followed by stiffness of the legs with internal rotation of the hips. She had suffered from multiple falls, even with radius and scaphoid fractures. Due to shortened muscles, myofasciotomy was performed on the hip adductors, knee flexors and calf muscles on both sides without any improvements in the symptoms. Neurological examinations revealed lower limb spasticity and muscle weakness with brisk deep tendon reflexes in both upper and lower limbs, a positive Babinski sign and a positive Romberg test. She had symmetrical atrophy of the lower leg muscles and foot muscles, and decreased vibration sense in both lower limbs. Electrophysiological examination revealed axonal, primarily sensory neuropathy. There was no dysarthria, dysmetria, intention tremor, muscle pain or abnormal eye movements. After the myofasciotomy, she experienced nocturnal muscle cramps and painful spasms, which were alleviated after taking baclofen. Additionally, she exhibited scoliosis, hyperhidrosis of the hands and feet and pes planovalgus on both sides. Cardiac involvement was not known prior to the investigation, and a follow-up cardiac examination had been recommended. An MRI scan of the brain was normal. Due to the age of onset, sensory loss in lower limbs, pyramidal signs and scoliosis, FRDA was one of the main differential diagnoses for the patient’s symptoms. The family history of neurological disease was unremarkable. She has one healthy half-sister and one healthy half-brother.

### 2.2. Genetic Analysis

Multiple genetic tests were performed from the age of 12 years, including an initial fragment analysis for FRDA together with a gene-panel analysis containing 121 genes associated with hereditary spastic paraplegia. This analysis revealed the known pathogenic missense variant (ENST00000484259.3:c.389G>T, p.Gly130Val) in *FXN* in a heterozygous state inherited from her healthy mother. However, the FRDA diagnosis could not be confirmed because the fragment analysis only showed normal alleles with approximately (GAA)16 repeats (Supplemental Figure S1A). Subsequently, a trio ES was performed at the age of 17 years, which did not reveal any other potentially disease-causing variant matching our patient’s phenotype. Only three years later, at the age of 20 years, a diagnostic short-read GS was performed, which detected a potential (GAA)n repeat expansion in *FXN* using the ExpansionHunter tool (Illumina Inc., San Diego, CA, USA) [13]. ExpansionHunter estimated a normal allele with (GAA)_17_ repeats (confidence interval [CI]: 17, 17) and an expanded allele of approximately (GAA)_112_ repeats (CI: 85, 189). To confirm and determine the repeat length, fragment analysis using TR-PCR and LR-PCR was repeated which again did not show any evidence of an expanded (GAA)n repeat allele. A very subtle, light band could be detected in the LR-PCR analysis at approximately 3600 bp (Supplemental Figure S1B).

**Figure 1:**
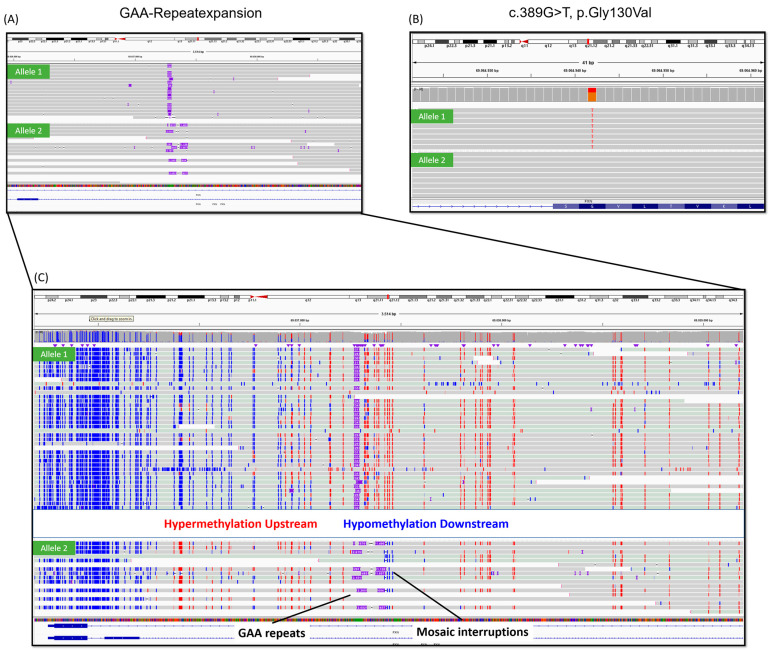
(**A**) Integrative Genomics Viewer (IGV). Screenshot of long-read genome sequencing (GS) of our index patient shows normal allele of (GAA)_17_ phased to one allele (Allele 1), and the expanded allele was phased to the other allele (Allele 2). (**B**) A heterozygous pathogenic missense variant was detected in our patient, which was phased to the allele carrying (GAA)_17_ repeats (Allele 1). (**C**) Base-modification 2-color 5 mc alignments show increased methylation (red) upstream and decreased methylation (blue) downstream of the repeat region.

**Figure 2:**
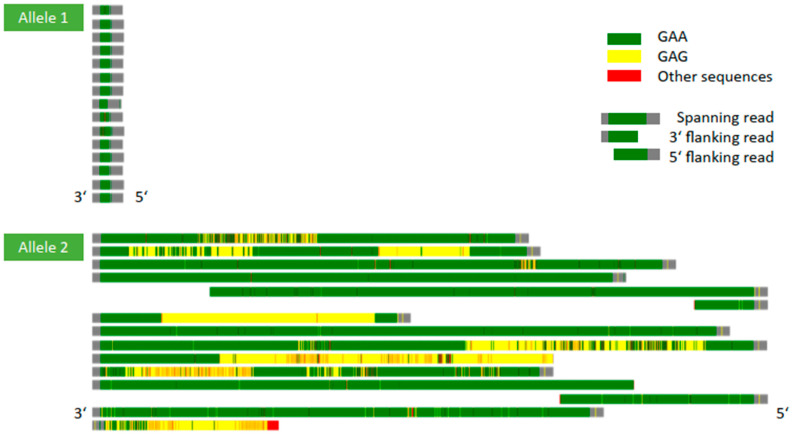
Graphical illustration of repeat motifs identified in each allele (15 example reads each). GAA triplets are shown in green, GAG triplets in yellow and other sequences in red. 3′ and 5′ sequences of *FXN* repeat region are depicted in grey. ONT sequencing identified variable lengths of (GAA)_146–1081_ repeats with different repeat interruptions, which contained (GAG)_64–504_ triplets and/or other combinations of single sequences (e.g., A, G, GA and AG).

A subsequent long-read GS detected both large (GAA)_839–1081_ repeats and smaller (GAA)_146–711_ repeats with divergent repeat interruptions. The smaller (GAA)_146–711_ repeats were interrupted by variable combinations of single G, GA, GAG or GAAG sequences. Some interruptions exhibited multiple (GAG)_64–504_ repeats with interspersed G-, A- and/or GA sequences, and one large interruption contained pure (GAG)_361_ repeats (Figure 1 and Figure 2). Large (GAA)_839–1081_ repeats also contained scattered A and GA interruptions (<1–3%). Three reads with small (GAA)_44–336_ repeats only partially covered the repeat expansion. When considering only spanning reads, the median GAA repeat length was 820 (IQR: 505–921; min–max: 146–1081). The *FXN* repeat region in intron 1 (hg38, chr9:69037286–69037304) had a coverage of 55× excluding reads with multiple artefacts, mismatched bases or various small deletions. Haplotype-phasing aligned reads containing the pathogenic missense variant and normal alleles with an average GAA repeat count of 17 to one allele (Figure 1 and Figure 2). Reads with (GAA)_n_ repeat expansions with different interruptions were phased to the other allele. Some reads remained unphased (seven with normal alleles, three with expansions). In total, 15 reads exhibited expansions (soft-clipped and fully-spanning), with the longest expansion (GAA)_1081_ illustrated as 3151 bp insertion in IGV. Long-read nanopore data were also used for profiling 5-methylcytosine (5mC) methylation, which detected high 5mC modification (illustrated as red in IGV) upstream and low 5mC modification (blue in IGV) downstream of the *FXN* repeat region in the repeat-expansion allele (Figure 1). In contrast, the normal allele exhibited low 5mC modification upstream and high 5mC modification downstream of the repeat area. Furthermore, the sequences flanking the repeat regions were investigated for small nucleotide polymorphisms (SNP) that may be incompatible with the primers used for fragment analysis (Supplemental Table S1). An intronic homozygous SNP on chromosome 9, g.69,037,043G>A (NM_000144.5:c.165+1096G>A), was detected within the LR-PCR forward primer binding site. Despite the SNP, the amplification of the normal allele was shown in the agarose gel of LR-PCR (Supplemental Figure S1), indicating that the primer binding sites are not accountable for the failed detection. Additional parental samples or other tissue samples were not available for long-read GS. The remaining parental DNA samples after trio exome sequencing did not have sufficient quality for long-read GS.

## 3. Discussion

To our knowledge, a mosaic sequence variation in a FRDA patient with somatic mosaicism with both large and small GAA expansions as well as divergent large interruptions has not been reported to date. The GAA repeat expansion could not be detected by conventional fragment analysis despite using both TR-PCR and LR-PCR. The amplification of the normal allele was present in the agarose gel of LR-PCR (Supplemental Figure S1), indicating that the SNP in the primer binding site is not accountable for the failed detection. The failure to reliably detect the expanded allele may be attributable to a combination of factors, including high somatic mosaicism, large size and complex secondary structures formed by interrupted repeats which may interfere with primer binding or polymerase processivity, even beyond the effect of a SNP. Nevertheless, ExpansionHunter was able to indicate the presence of a heterozygous repeat expansion based on short-read GS. Ultimately, we identified and determined the GAA repeat length using long-read GS, which detected complex interruptions mostly containing GAG repeats and other simple tandem repeat variations (e.g., G, A, AG or GA). Furthermore, the expanded allele showed characteristic hypermethylation in the upstream region of GAA repeats consistent with the pathomechanism of FRDA.

The FRDA GAA triplet repeats are known to show intergenerational and somatic instability [11,14]. Somatic mosaicism within and between different tissues has been reported to hamper the accurate estimation of repeat length [11]. Because disease onset and progression strongly correlate with the repeat length, this value is of great interest to patients, family members and clinicians. Large interruptions of GAA repeat expansions are rare among FRDA patients [14]. Al-Mahdawi et al. have attempted to specifically address the question whether such interruptions can show intergenerational or somatic changes by examining four carrier parents and different tissue samples from three patients. In this small subset of selected patient cases, no large repeat interruptions could be detected [14]. The role and pathogenicity of such complex interruptions is still debated: it has been postulated that interrupted repeats are more stable, potentially delaying age of onset and reducing the severity of disease [7,11,14]. However, such large and complex interruptions with high disparities in a single allele have not yet been reported.

Some limitations that need to be addressed are the lack of other tissue samples from our patient as well as additional parental samples for subsequent analyses. Tissue-specific somatic instability and variability in GAA repeat lengths have been observed in some FRDA patients [11]. This may contribute to differences in disease onset, severity and progression and may explain the clinical variability in patients with comparable repeat lengths in blood. FRDA patients did not only harbor variable repeat lengths, but also different frataxin expression levels in different tissue samples [11]. Since complex mosaicism is already present within blood leukocytes, the somatic instability may also be present in different tissue samples. As we were able to detect hypermethylation, like in other patients with elongated repeats, we assume that the complex alteration also leads to reduced frataxin expression in our patient, which may also vary in different tissues. In addition, our patient may have inherited an instable expanded allele from her father. A technical limitation of native long-read GS is the lower coverage of repeat regions, in which somatic mosaic variations may be underrepresented, as demonstrated in our case. Targeted long-read sequencing which can yield very high depths of coverage may be advantageous for the detection of somatic sequence variations. The feasibility of successful targeted long-read sequencing in FRDA patients has recently been published by an Indian group [15]. They also demonstrated that GAA repeats were often interrupted by GGAGAA or multiple dispersed single A and GA sequences [15]. Large GAA repeats contained scattered small A and GA interruptions or other indels in 1–8% of sequences, possibly attributed in part to sequencing artifacts associated with ONT [15]. These minor sequencing artifacts were also observed in the repeat region of our patient.

Somatic mosaicism and repeat interruptions also occur in other repeat disorders [16,17,18]. These observations have led to longstanding questions regarding their potential contribution to the wide clinical variability and atypical clinical courses, which yet remains to be further elucidated. While repeat expansion detection tools such as ExpansionHunter in SR genome sequencing shows excellent performance in detecting short STR, it reaches its limits when it comes to very long or structurally complex repeat structures. As short reads are not able to capture the long repeats as spanning reads, an accurate estimation of the size can be difficult. Long-read sequencing technologies of PacBio or ONT have been utilized to accurately determine repeat lengths, the level of mosaicism and repeat interruptions in patients with other repeat disorders such as Fragile X [16], myotonic dystrophy [17] and X-linked dystonia-parkinsonism [18]. These long-read sequencing studies have so far only included patients that had already been diagnosed with the corresponding repeat disorders. The increasing use of long-read GS has also revealed numerous new disease-associated repeat expansion loci in recent years, with over 50 repeat disorders reported to date [19]. With the constantly growing number, it is becoming more difficult to establish and conduct targeted testing for all repeat disorder genes. Especially in cases with atypical clinical presentations, there is a chance of missing the diagnosis.

In this study, we present a FRDA case exhibiting somatic mosaicism and various large interruptions within a single *FXN* allele. Our case underscores the limitations of conventional methods when dealing with complex genomic variations. Long-read GS emerges as a valuable tool to overcome these limitations, uncovering genetic variations that may otherwise remain undetected. The presence of clinical features of FRDA, a pathogenic missense variant in one allele and an expanded allele displaying the characteristic methylation pattern facilitated an accurate molecular diagnosis more than 10 years after the onset of symptoms. Further identification and characterization of such complex interruptions and somatic instability in additional FRDA patients are needed to shed more light on their contribution to the clinical course.

## 4. Materials and Methods

Our patient was evaluated by neurologists and referred for diagnostic exome sequencing (ES) at the age of 17 years and short-read GS at the age of 20 years. DNA was extracted from blood leukocytes. The sample for ES was processed with the SureSelectXT Human All Exon v6 kit (Agilent, Santa Clara, CA, USA) and sequenced on a NovaSeq6000 system (Illumina, San Diego, CA, USA) as 100 bp paired-end reads with a target depth of 150x. The sequencing library for short-read GS was generated using the TruSeq DNA PCR-Free kit (Illumina) and sequenced on a NovaSeq6000 system as 150 bp paired-end reads with an average coverage of 34×, as previously published [20]. For long-read GS, libraries were generated from genomic DNA using a 1D Ligation LSK114-XL Sequencing kit V14 and a PromethION R10.4.1 flow cell (Oxford Nanopore Technologies [ONT], Oxford, UK). Sequencing was performed on a Nanopore PromethION 24 (Oxford Nanopore Technologies) as previously published [21]. The sequencing reads were basecalled on the device with dorado (v0.5.3) through the MinKnow application using the ‘dna_r10.4.1_e8.2_400bps_hac@4.2.0′ model for DNA and the ‘5mCG_5hmCG@v2’ model for base modification detection. Reads were mapped to the GRCh38 reference genome using minimap2 (v2.26) with the ‘map-ont’ parameter preset and phased with longphase (v1.7.3) after variant calling with Clair3 (v1.0.2). All analysis steps were run as part of the megSAP pipeline (https://github.com/imgag/megSAP, accessed on 08.11.2023) [22,23,24]. The average sequencing depth in the target region was 63×, and the N50 read length was 13,780 bp for the reported sample. The PCR conditions and primers used for TR-PCR and LR-PCR are summarized in Supplemental Table S1. Written informed consent for publication was obtained from o169 + 347ur patient according to the Declaration of Helsinki.

## Data Availability

Data available upon reasonable request due to restrictions (privacy reasons).

## Supplementary Materials

The following supporting information can be downloaded at: https://www.mdpi.com/article/10.3390/ijms26114969/s1.

## Abbreviations

The following abbreviations are used in this manuscript:

FRDA	Friedreich’s ataxia
GS	Genome sequencing
STR	Short tandem repeats
TR-PCR	Triple-repeat-primed PCR
LR-PCR	Long-range PCR
