# Supplementary material for: Long-Read Sequencing Identifies Mosaic Sequence Variations in Friedreich’s Ataxia-GAA Repeats
Source: Int J Mol Sci. 2025 May 22;26(11):4969. doi: 10.3390/ijms26114969 (PMC12154355; doi:10.3390/ijms26114969)
Supplement: Supplementary file 1 [file ijms-26-04969-s001.zip › ijms-3609266-supplementary.pdf]

Supplemental Table S1: PCR conditions and primers used for TR-PCR and LR-PCR

Triple-repeat primed PCR (TR-PCR)		
Primers:<br>Forward: 5’-GTGGCTCATGCCCATAATCT-3’<br>Reverse: 5’-GCCCGGCTAACTTTTCTTTA-3’		
Expand Long Range PCR System (Roche)	1 x 50µl	Slow mode
H2O	26,6	5min/94 °C
10x PCR-Buffer 1	5µl	36x
dNTPs	1µl	30 s/94 °C
Q-Solution 5x	10µl	30 s/56 °C
Taq	0,4µl	30 s/72 °C
Primer	4µl	10 min/72 °C
DNA/cDNA	3µl	∞ /10 °C

Repeat specific Primers		
Forward1: 5’-GCTGGGATTACAGGCGCGCGA-3’<br>Reverse: 5’-TACGCATCCCAGTTTGAGACGGAAGAAGAAGAAGAAGAAGAA-3’,<br>Forward2: 5’-TACGCATCCCAGTTTGAGACG-3’		
FastStart Master Mix (Roche)	1 x 40µl	Slow mode
H2O	7,68µl	5 min/94 °C
10 x PCR-Buffer	4µl	10x
dNTP´s - Mix	2µl	30 s/94 °C
Betain (200mM)	8µl	30 s/65°C -1°C
Q-Solution	8µl	30 s/72 °C
Taq	0,32µl	35x:
Primer	4µl	30 s/94 °C
DNA/cDNA	6µl	30 s/55 °C
		30 s/72 °C
		7 min/72°C
		∞ 10°C

Long Range PCR Primers		
Forward: 5’- GGCTTAAACTTCCCACAGTGTT-3’<br>Reverse: 5’- AGGACCATCATGGCCACATT-3’		
UltraRun® LongRange PCR Kit (Qiagen)	1x20µl	Fast mode
H20	7µl	3 min/93°C
UltraRun Longrange PCR Master Mix, 4x	5µl	30x
Q-Solution,5x	4µl	30s/93°C
Primer mix (10pmol/µl)	1µl	15s/55°C
DNA	3µl	10min/68°C
		10min/72°C
		∞ 10°C

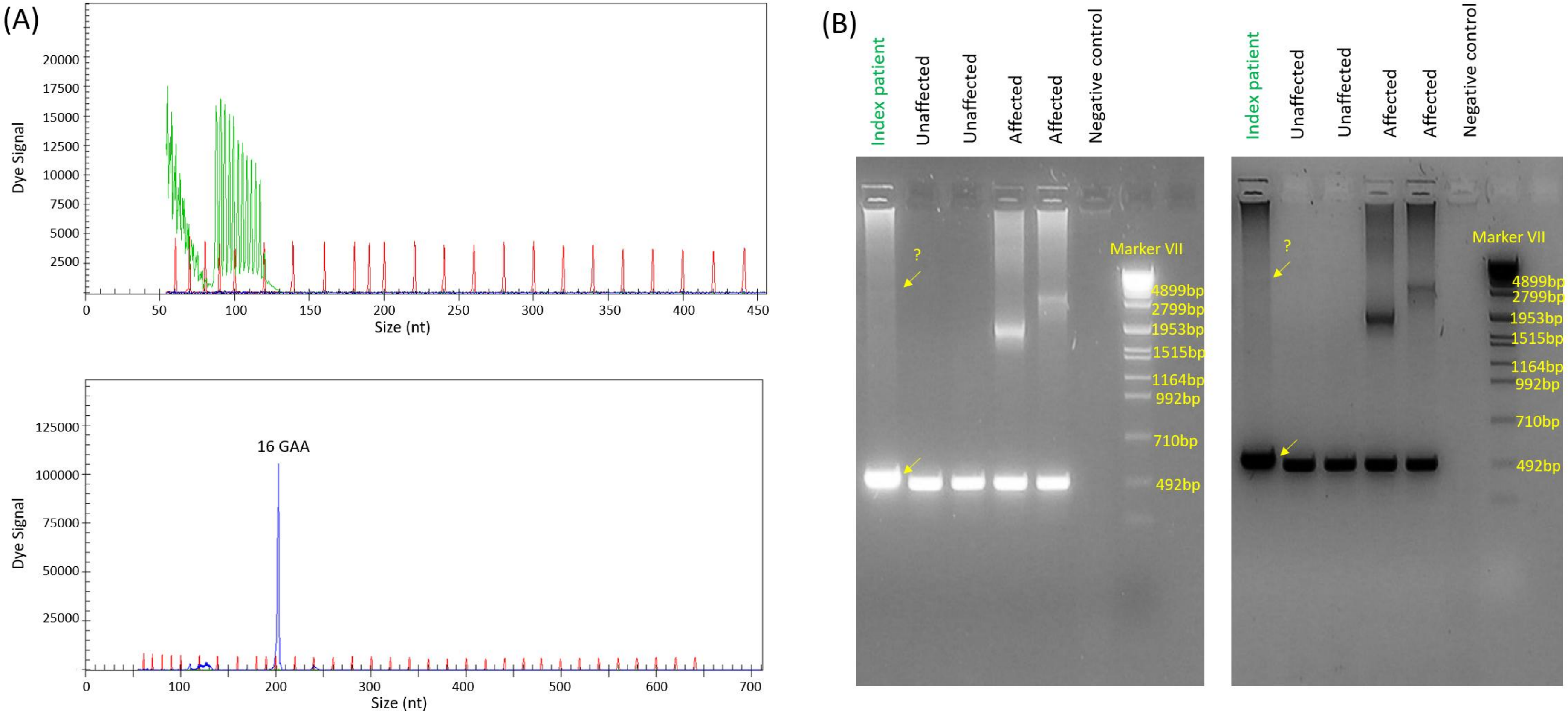
(A)
Dye Signal
Size (nt)
16 GAA
(B)
Index patient
Unaffected
Unaffected
Affected
Affected
Negative control
Marker VII
?
4899bp
2799bp
1953bp
1515bp
1164bp
992bp
710bp
492bp
